# Identifying Archaeological Bone via Non-Destructive ZooMS and the Materiality of Symbolic Expression: Examples from Iroquoian Bone Points

**DOI:** 10.1038/s41598-019-47299-x

**Published:** 2019-07-30

**Authors:** Krista McGrath, Keri Rowsell, Christian Gates St-Pierre, Andrew Tedder, George Foody, Carolynne Roberts, Camilla Speller, Matthew Collins

**Affiliations:** 1 BioArCh, Department of Archaeology, University of York, York, UK; 2 London School of Economics and Political Science, London, UK; 3 Département d’anthropologie, Université de Montréal, Montréal, Québec Canada; 4 School of Chemistry & Bioscience, University of Bradford, Bradford, UK; 5 Department of Biological and Geographical Sciences, School of Applied Sciences, University of Huddersfield, Huddersfield, UK; 6 Department of Anthropology, University of British Columbia, Vancouver, Canada; 7 EvoGenomics, Natural History Museum of Denmark, University of Copenhagen, Copenhagen, Denmark; 8 McDonald Institute for Archaeological Research, University of Cambridge, Cambridge, UK

**Keywords:** Haplotypes, Proteomics

## Abstract

Today, practical, functional and symbolic choices inform the selection of raw materials for worked objects. In cases where we can discern the origin of worked bone, tooth, ivory and antler objects in the past, we assume that similar choices are being made. However, morphological species identification of worked objects is often impossible due to the loss of identifying characteristics during manufacture. Here, we describe a novel non-destructive ZooMS (Zooarchaeology by Mass Spectrometry) method which was applied to bone points from Pre-Contact St. Lawrence Iroquoian village sites in southern Quebec, Canada. The traditional ZooMS technique requires destructive analysis of a sample, which can be problematic when dealing with artefacts. Here we instead extracted proteins from the plastic bags in which the points had been stored. ZooMS analysis revealed hitherto unexpected species, notably black bear (*Ursus americanus*) and human (*Homo sapiens sapiens*), used in point manufacture. These surprising results (confirmed through genomic sequencing) highlight the importance of advancing biomolecular research in artefact studies. Furthermore, they unexpectedly and exceptionally allow us to identify and explore the tangible, material traces of the symbolic relationship between bears and humans, central to past and present Iroquoian cosmology and mythology.

## Introduction

In human societies, animals are entangled and hybrid objects (sensu^1^), both material objects and creators of identity. Social zooarchaeology^2^, a component of the so called ‘animal turn’ in social sciences and humanities^3^, problematizes this human-animal divide and seeks to understand how animals were integrated into the social and ideological lives of humans in the past. For example, ethnographic studies of indigenous groups^4–8^ reveal that hunters perceive animals as other-than-human persons who have agency, morals, and responsibilities.

Archaeologists have access to the material culture associated with hunting but lack such direct ethnographic insights into cosmologies^9^. Much archaeological investigation of such material culture has focused on the functionality and accessibility of the material (e.g. stone requires less investment than bone^10^). However, in crafting objects, ancient artisans would have been influenced not only by the shape, strength, plasticity, and availability of osseous materials^10^, but also other factors such as prestige or symbolism^11^. The more highly crafted a bone tool, the more difficult it is to identify the species from which it was made. Conversely, the more highly worked such an object is, the less likely permission will be given for destructive biomolecular analysis in order to identify species^12^. Thus, archaeologists are frustratingly aware of the resonant power of a materiality they cannot access. This can be illustrated by the extensive literature on the use of human bone as raw material. Reports of worked human bone tend to describe objects which have meaning in remaining recognisably human (e.g. skull cups^13^).

Our study sought to highlight the multiple roles of bones in the material culture of Iroquoian societies, where animals are used and conceived as sources of food, raw materials and symbolic expressions. Originally, it was believed that raw material selection from three Pre-Contact Iroquoian village sites located in southern Quebec, Canada, would have largely been a function of size and availability. Since the distinctive beveled projectile points from the sites appear to be rather standardised, it was hypothesised that they were made using the bones of a single species. White-tailed deer appeared as the most probable candidate, based on its abundance in the fish-dominated faunal assemblages, as well as the size of its long bones that could have served as production blanks. Problematically, the highly worked conical bone projectile points were stripped of identifiable species characteristics, whilst at the same time their aesthetic appeal increased our reluctance to perform destructive analysis.

We report on a breakthrough identification method, a wholly non-destructive analysis of an object-at-distance, by analysing bone proteins from the bags in which objects are stored. The Iroquoian bone points had been stored in zip-seal bags, and it was reasoned that the tool rubbing against the polyethylene of the bag could generate a triboelectric charge which could be exploited in a similar manner as the previously reported polyvinyl chloride (PVC) eraser rubbings used to identify the animal origin of parchment^14^. Both the original storage bags and new, previously unused bags were tested, and the results compared to those of the aforementioned eraser method. The identifications resulting from the non-destructive methods were subsequently confirmed using destructive proteomic and genomic analyses.

## Archaeological Setting

**Figure 1:**
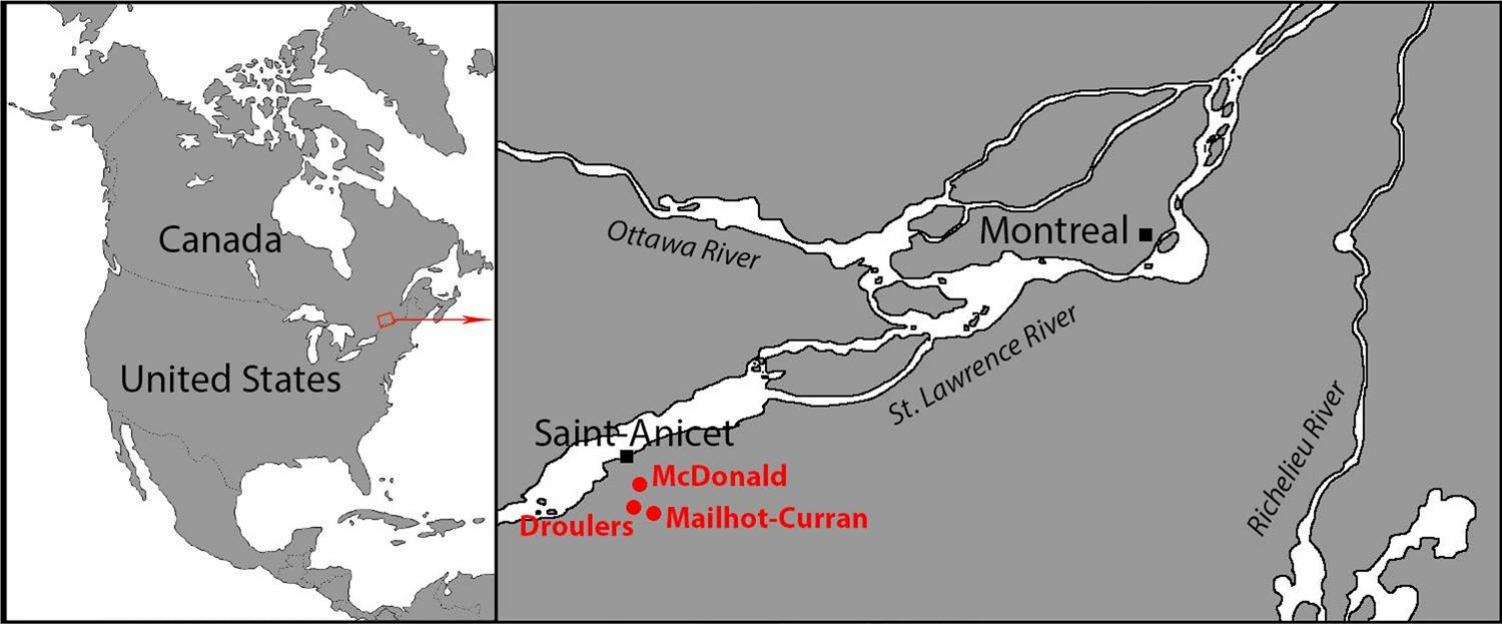
Location of the three St. Lawrence Iroquoian village sites (Droulers, McDonald and Mailhot-Curran) discussed in this study.

The artefacts in question were excavated from the Droulers, McDonald and Mailhot-Curran sites, three St. Lawrence Iroquoian village sites dating to the middle of the 14th to the late 16th centuries AD, located roughly 75 km southwest of Montreal (Fig. 1). Large quantities of faunal remains, including hundreds of complete and fragmented bone tools and manufacturing debris, were recovered from excavations spanning the early 1990s to 2017^15–18^. Fish remains heavily dominate the faunal assemblages in proportions varying from 69% (McDonald) to 96% (Droulers), with yellow perch (*Perca flavescens*) being by far the most important species. The most abundant identified mammal remains are those of white-tailed deer (*Odocoileus virginianus*), followed by beaver (*Castor canadensis*)^17,18^. Other medium to large mammals are also present, such as snowshoe hare (*Lepus americanu*s), muskrat (*Ondatra zibethicus*), and black bear (*Ursus americanus*), but in proportions of less than 5% of the total mammal bones morphologically identified.

**Figure 2:**
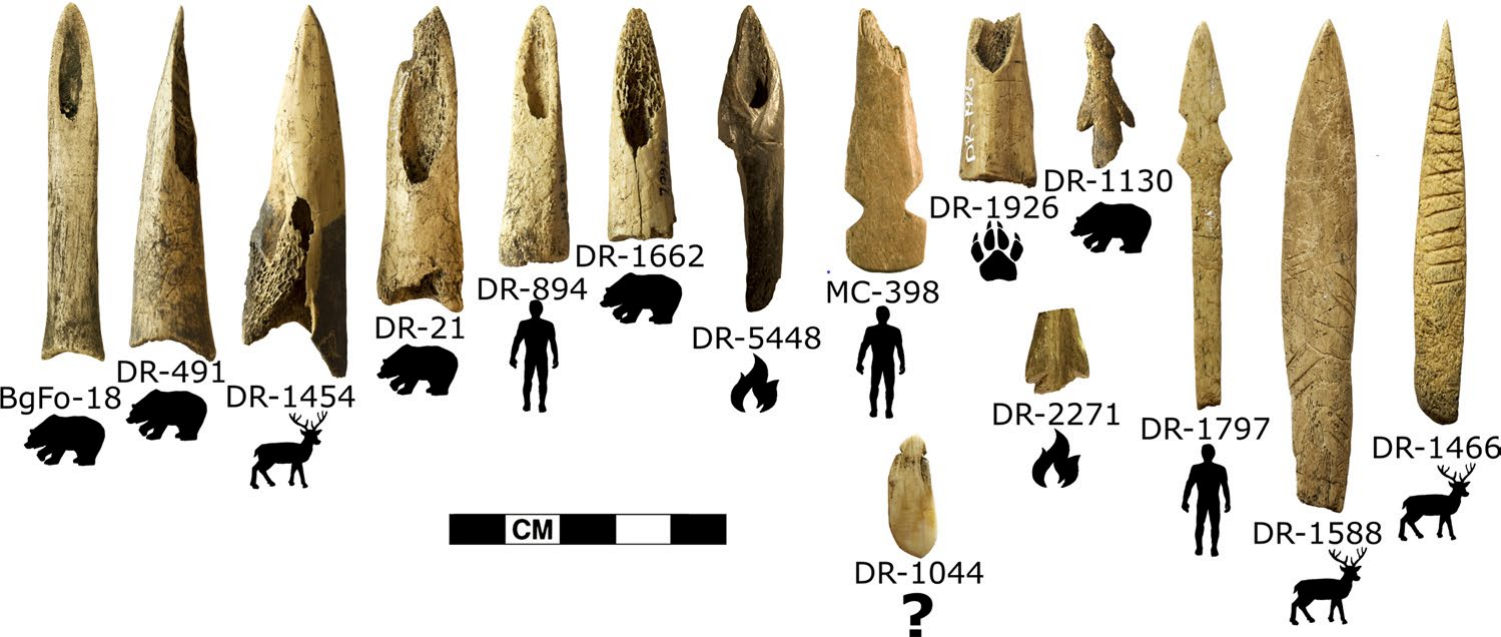
Bone artefacts analysed from the Droulers (DR-), McDonald (BgFo-) and Mailhot-Curran (MC-) sites, with ZooMS identification of bear, human or deer indicated. Fire symbol denotes burnt samples; paw print denotes unknown carnivore; “?” denotes unknown ID.

A wide variety of bone tools and objects were recovered, including awls, projectile points, harpoon heads, needles, chisels, flakers, beads, pendants, and pieces of the cup-and-pin game^17,18^. The projectile points include a recently defined type that appears to be specific to the St. Lawrence Iroquoians, characterised by a conical shape and a beveled distal end^16^. Seven points of this type, along with eight other bone artefacts (Fig. 2), were selected for species identification via ZooMS (Zooarchaeology by Mass Spectrometry).

### Methods context

ZooMS was first proposed by Buckley *et al*.^19^ as a method for identifying the species of bone fragments where no morphological indicators are present. The method uses peptide mass-fingerprinting of Type I collagen through Matrix Assisted Laser Desorption/Ionisation Time of Flight Mass Spectrometry (MALDI-ToF-MS). ZooMS is widely used in archaeology and paleontology, with an expanding range of applications due to newly sequenced collagen genes from multiple species (e.g.^20–26^). Nevertheless, the method of collagen isolation - a variant on that used to isolate collagen for stable isotope and radiocarbon dating - is destructive. The bone mineral is removed by acid demineralisation, and the residual collagen ghost is gelatinised, prior to enzyme digestion and peptide analysis.

The destructive nature of ZooMS is undesirable when analysing complete or rare artefacts made from bone yet it is precisely these artefacts, which have had much of their identity stripped from them, for which ZooMS offers the greatest potential. There are several important considerations when deciding whether to destructively sample an artefact, including likelihood of analytical success, choice of sampling technique to ensure minimal structural and visible effects on the object, quantity of material to sample, and effects of current sampling on future research. A biomolecular method of species identification that will not damage the object, which leaves little or no visible traces on the object, and which has no knock-on effects for potential future work or collection curators, could enable museums to open their collections to a greater number of academic researchers. This is especially pertinent when considering the potential information that could be gained regarding rare artefacts of particular importance.

It was the previously reported non-destructive ZooMS technique using the triboelectric effect of a PVC eraser, initially established for the analysis of parchment^14^ and now being tested on other archaeological materials such as bone and ivory^27^, which drove us to consider the storage bags. Frictional contact of plastic (triboelectric negative) with protein (triboelectric positive) strips electrons from protein and results in charge electrification, and allows loose protein molecules to be pulled away from the bone. As the bone artefacts were stored in polyethylene zip-seal storage bags (as is commonly the case), we reasoned that they had undergone similar charge electrification, and therefore attempted to remove the adhering collagen from the plastic surface.

## Results

### ZooMS analysis: non-destructive vs destructive methods

We applied four different ZooMS approaches to the bone artefacts: collagen extraction from the original storage bag surface (original bag method); collagen extraction from a new storage bag surface following gentle manipulation of the artefact within (forced bag method); collagen extraction from the artefact using a PVC eraser (eraser method)^14^; and the conventional destructive ZooMS method (destructive ZooMS)^19^. The forced bag method was developed to resolve two potential problems: (1) storage bags may have been used for multiple samples; and (2) degradation of the collagen fragments adhering to the bags due to the use of HCl and NaOH in the original bag method. The original bag method involved an initial acid wash designed to demineralise any tiny pieces of bone that may have remained in the bags, followed by NaOH to neutralise the HCl. We removed these steps in the forced bag method as the benefits of potentially demineralising any small bone pieces were outweighed by several downsides of using the acid, i.e., the buildup of salt during neutralisation (which is known to interfere with downstream analysis), and poor quality spectra likely due to acid-induced damage to the adherent collagen. The forced bag method solved both aforementioned issues by manipulating the artefact in a new, previously unused storage bag, followed by extraction using an ammonium bicarbonate buffer (AmBic) instead of acid. These modifications resulted in improved spectra and increased identifications (to varying taxonomic levels) (Table 1).

**Table 1:** Species/genus identifications of the 15 bone artefacts for each of the ZooMS methods tested, and subsequent DNA identifications.

Sample	Artefact Type	Original Bag	Forced Bag	Eraser	Destructive	DNA
DR-21s	Bevelled conical point	Bear	N/T	Probable bear	Bear	*U*. *Americanus*
DR-491s	Bevelled conical point	Probable bear	Probable bear	Probable bear	Bear	N/T
DR-894s	Bevelled conical point	X	N/T	Human	Human	*H*. *sapiens*
DR-1044s^1^	Pendant	N/T	N/T	X	N/T	N/T
DR-1130s	Harpoon	X	X	Carnivora (possible Cat/Bear)	Bear	N/T
DR-1454s	Point	Probable Bovid/Cervid	Probable Bovid/Cervid	Probable Bovid/Cervid	White-tailed deer	N/T
DR-1466s	Point or awl	Probable Bovid/Cervid	Probable Bovid/Cervid	Probable Bovid/Cervid	White-tailed deer	N/T
DR-1588s	Point or awl	X	Probable Bovid/Cervid	Probable Bovid/Cervid	White-tailed deer	N/T
DR-1662s	Bevelled conical point	Probable Bear	N/T	Bear	Bear	*U*. *Americanus*
DR-1797s	Harpoon	X	Human	Human	N/T	*H*. *sapiens*
DR-1926s	Bevelled conical point	X	Carnivora	Carnivora	Carnivora	N/T
DR-2271s^2^	Harpoon	X	N/T	X	N/T	N/T
DR-5448s^2^	Bevelled conical point	X	N/T	X	N/T	N/T
MC-398s	Point	X	Human	X	Human	Fail
BgFo-18	Bevelled conical point	X	X	Carnivora	Bear	N/T
Control bag	N/A	X	X	N/A	N/A	N/T

Note: X indicates no identification could be made; N/T indicates the method was not tested on the arfefact; N/A indicates not applicable to the method. 1 - tooth pendant; 2 - burnt samples.

We performed at least one ZooMS method on each of the 15 artefacts, subjecting the majority of samples to all methods (Table 1). In order to confirm the non-destructive identifications and to potentially provide greater taxonomic resolution, we then applied destructive ZooMS to 11 of the bone artefacts. DR-2271s and DR-5448s were heavily burnt and did not yield any results from either non-destructive method. Burning is known to significantly degrade collagen in bone^28^, and given the extent of the burning in both specimens, destructive analysis was deemed unlikely to yield surviving collagen. DR-1797s was identified as human through both non-destructive ZooMS methods, as such destructive ZooMS was deemed unnecessary and further destructive analysis was conducted only for genomic analyses. Finally, only the eraser method was used for DR-1044s - a pendant originally believed to have been made of either highly polished bone or ivory - as the specimen had been wrapped in tissue inside the original storage bag, preventing any residual collagen transfer. Due to its delicate nature, destructive sampling was not performed, however microscopic analysis determined it was manufactured from a tooth (likely Artiodactyl based on size and shape), rather than bone. As the highly polished surface of DR-1044s suggested it was manufactured from enamel (which does not contain collagen) and since it failed to yield results using the eraser method, we elected not to test this artefact using the forced bag method.

The 11 artefacts that underwent destructive ZooMS were all identified to the species or family level, with the exception of sample DR-1926s which could only be identified as likely belonging to the Order Carnivora (Table 1, SI Table 2). As DR-1926s yielded spectra of good quality for all ZooMS methods, the failure to resolve a more precise identification for it reflects a gap in our current knowledge of the collagen sequences of North American mammals, rather than analytical failure of the various ZooMS methods. We confronted a similar lack of data for North American deer, such as white-tailed (*O*. *virginianus*) and mule (*O*. *hemionus*) deer, with only a single published Type I collagen sequence (transcribed from genomic data) for white-tailed deer available on NCBI^29^. Based on the species local to the study area and present within the zooarchaeological assemblages, we assumed the three bone artefacts identified by ZooMS as “deer” were white-tailed deer, and subsequently confirmed these identifications through comparison with spectra obtained from comparative reference material (Table 2 and SI Table 2).

**Table 2:** Designated *m/z* markers for taxonomic identification of five cervid species, and the three artefacts identified as “deer” in this study.

*m/z* marker	Roe Deer	Red Deer	Fallow Deer	Caribou/ Reindeer	White-Tailed Deer	DR-1454s	DR-1466s	DR-1588s
1105	P1	P1	P1	P1	P1	P1	P1	P1
1150 + 1166				A				
1180 + 1196	A	A	A		A	A?	A	A
1427	B	B	B	B	B	B	B	B
1550	C	C	C					
1580				C	***C***	*—*	***C?***	***C***
1648	P2	P2	P2	P2	P2	P2	P2	P2
2131	D	D	D	D	D	D	D	D
2883 + 2899	F	F	F	F	F	F?	F?	F
3017 + 3033		G	G					
3043 + 3059	G				***G***	***G***	***G***	***G***
3093				G				

? Indicates peak is present but at low intensity, or below signal to noise threshold. The presence/absence of a particular peptide marker is denoted by letters P1, P2, A-G; A = α2(I) 988–1000; B = α2(I) 494–508; C = α2(I) 512–529; D = α2(I) 803–826; F = α1(I) 602–634; G = α2(I) 767–799. “—” Indicates no peak was present. Where two *m/z* values are given, the presence of both is required. Bold italicised text indicates species specific markers. With the exception of the white-tailed deer and the three DR samples, the cervid *m/z* markers are from published sources^19,49,50^. The white-tailed deer *m/z* markers were determined from spectra obtained from a known reference specimen.

### Ancient genomic analysis

Due to the unexpected diversity of species used to manufacture the bone artefacts, in particular the identification of bears and humans within the assemblage, we sought to validate the ZooMS identifications and produce population-level resolution through genomic analysis. We extracted DNA and performed shotgun sequencing on five bone points (two bears (DR-21s and DR-1662s) and three humans (DR-894s, DR-1797s and MC-398s)). FastQ Screen analysis (https://www.bioinformatics.babraham.ac.uk) of the trimmed and quality filtered genomic sequences identified bear as the most likely source for the majority of the sequences from DR-21s and DR-1662s, and human as the most likely source for DR-894s and DR1797s, though no dominant genome could be determined for MC-398s (SI Fig. 1). The genomic sequences, therefore, broadly validate the ZooMS identifications.

We further resolved the identification of the two ursid points (DR-21s and DR-1662s) through targeted amplification of mitochondrial DNA fragments (Supplementary Information S2). We amplified and aligned 215 bp of the cytochrome b gene to available reference sequences from black bear (*Ursus americanus*), brown bear (*Ursus arctos*), polar bear (*Ursus maritimus*), and the now extinct short-nosed bear (*Arctodus simus*). Both DR-21s and DR-1662s were identified as black bear (*U*. *americanus*), grouping with A-East haplotypes, consistent with the bear species and haplogroup in the study region^30^.

**Figure 3:**
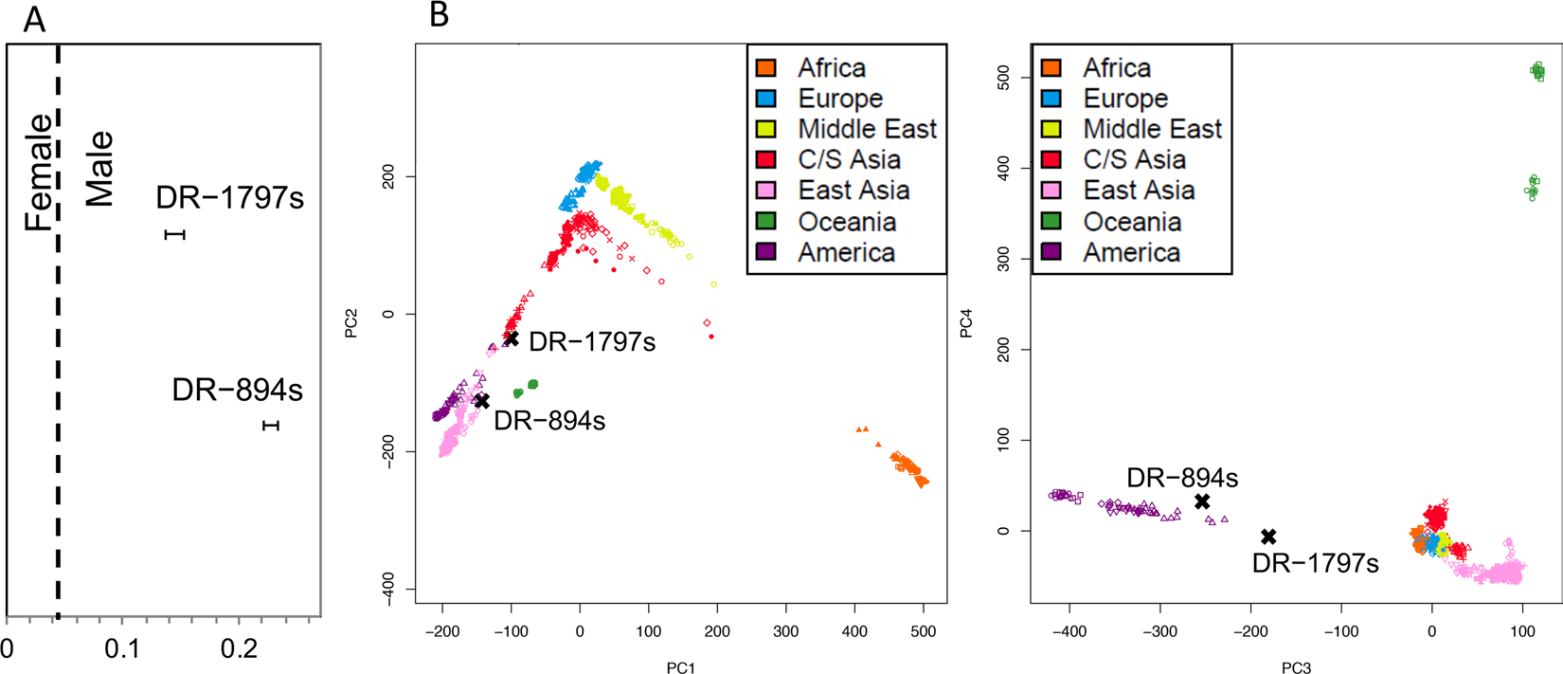
Results of genomic analysis of human bone points. (**A**) Ratio of genomic reads aligning to the Y chromosome to reads aligning to both sex chromosomes (Ry) indicating the male sex of both individuals. (**B**) Procrustes transformation of Principal Component Analysis combining the two human bone point samples with 938 modern humans from the Human Genome Diversity Panel indicating the affinity of the two bone points with indigenous American populations.

We performed whole genome sequence analysis to further investigate the origin of the human bone points. Human bone point MC-398s failed to yield sufficient human DNA for genomic analysis. Although the endogenous human DNA content of both DR-894s and DR-1797s was relatively low (2.9% and 2.2%, respectively, SI Table 1), molecular sex identification indicated that both points derived from male individuals (Fig. 3). We used a projection principal component analysis (PCA) to investigate the genetic affinities of DR-894s and DR1797s, alongside 938 modern individuals from the Human Genome Diversity Panel^31^ (Fig. 3). We obtained a limited number of SNPs for DR-894s and DR-1797s (7585 and 3094 SNPs, respectively). Within the first two dimensions of the PCA, DR-894s groups with modern populations from the Americas, while DR-1797s groups with modern populations from East and South/Central Asia, as well as the Americas. The third dimension of the PCA more effectively distinguishes Native American populations from other world-wide groups. Here, both samples show a greater affinity to modern Native Americans than to individuals of East and South/Central Asian ancestry, with DR-1797s drifting towards the mean, most likely due to low SNP coverage (although low-level contamination cannot be excluded as a factor). Sufficient mitochondrial DNA sequences were obtained from DR-894s to assign a mitochondrial haplogroup, detecting haplogroup C1, one of the founding haplogroups of the Americas. In spite of the limited DNA recovery, the combined genomic results are most consistent with a local (i.e., Indigenous American) origin for both individuals.

## Discussion

Our study aimed to determine the extent to which accurate taxonomic identifications could be obtained through non-destructive analysis of highly worked bone objects. While higher-order identifications were obtained for the majority of the artefacts using non-destructive techniques, destructive analysis was required to clarify species level identifications. Nevertheless, all methods rejected our initial hypothesis of deer bone as the most common source material for bone tool manufacture, instead identifying unexpected species of high cultural importance and significance, namely human and bear. Here, we discuss the cultural significance of these identifications, and highlight the pitfalls and potentials of applying this newly developed non-destructive method to highly-worked or culturally significant biological artefacts in other repositories.

Black bear is often identified in bone assemblages from Iroquoian sites, but it is never dominant. Bones from other mammals such as white-tailed deer, beaver, groundhog, or canids are usually much more numerous, suggesting that the economic value of bear is somewhat limited. The symbolic value of bear is more fundamental, however. As Levi-Strauss would say, bears are not important animals because they are good to eat, but because they are good to think^32^.

American anthropologist A. Irving Hollowell long ago identified the prime importance of bear in aboriginal cosmologies of the circumpolar area, especially northern North America and Asia^4,33^. This has also been documented for Iroquoian societies of the Eastern Woodlands of North America through archaeological, ethnographic, and ethnohistorical records^34–38^. In many cases they testify to the very close link that existed, and still exists, between humans and bears, that “other-than-human being”. In order to get closer to, emulate, or even acquire the highly valued aptitudes and qualities of bears, Iroquoian societies have developed symbolic ties with them, which can take many forms. For example, bear was frequently chosen as an emblem, hence the common “clan of the bear” ethnozoonym among Iroquoian societies^38–40^. Similarly, bears were often given familial names such as “brother” or “grandfather”^4,41^. This human-animal relationship served as an ontological means for some Iroquoians to define and situate themselves in the natural and spiritual world.

The symbiotic and symbolic relationship between humans and bears is also clearly expressed in aboriginal myths and legends, including those of the Iroquois^35–37^. Hallowell^4^ again made a convincing demonstration of this, and it is especially interesting to note that many such stories illustrate interspecies relations and transformations. In some Iroquois myths and legends, humans come to live like bears after their adoption by the latter^35,36^, while the Huron and Wyandot also had their own versions of these^37^.

As archaeologists, the challenge is to find convincing material evidence of this symbolic entanglement between humans and bears. Betts *et al*.^42^ provide a rare, yet convincing case in their analysis of bear figurines from the Dorset culture of the Canadian Arctic. The case at hand is another example of the materialisation of the defining relationship between humans and bears. As we have seen, most of the bone artefacts in our sample that were used for hunting – projectile points and harpoon heads – were made with human or bear bones, including a group of seven projectile points of the bevelled and conical type that appear to be characteristic of the St. Lawrence Iroquoians^16,43^. While scattered human remains are not rare on Iroquoian sites^44–47^, human bones were rarely used as a raw material to produce artefacts. In the present case, the preferential selection of human and bear bones is quite telling. Bear and human bones appear to have been deliberately chosen to materially express their mutual entanglement, to symbolically transpose the hunting skills of bears into the hands of humans using these bone points to kill other beings, whether human or animal.

**Figure 4:**
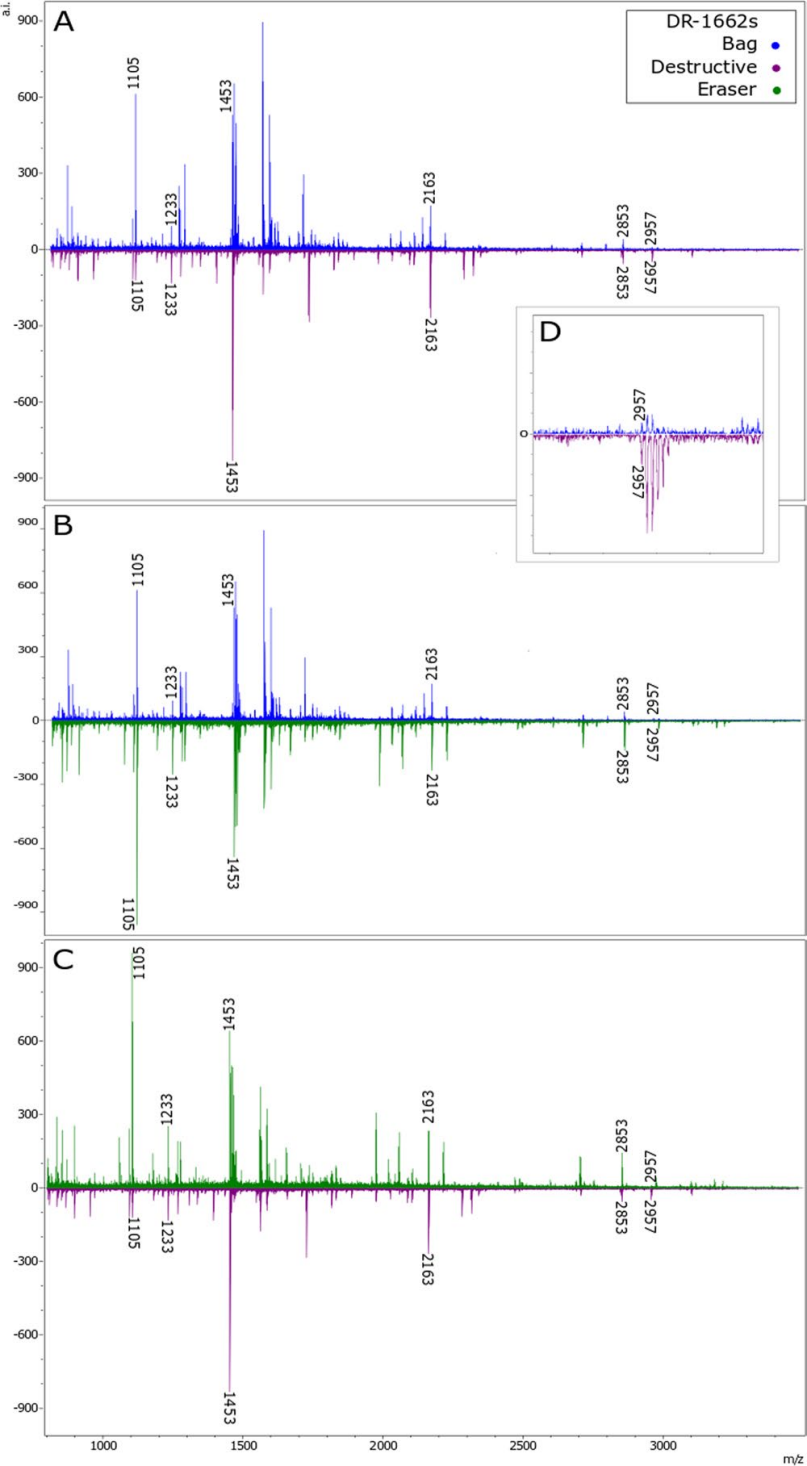
Comparison of MALDI-ToF-MS spectra from the bag, eraser and destructive ZooMS methods for sample DR-1662s, showing peaks used to identify it as bear. (**A**) Bag vs destructive; (**B**) bag vs eraser; (**C**) eraser vs destructive; (**D**) close up of spectra in (**A**) showing poor resolution of high molecular weight peptides in the bag compared to the destructive method. Bag method spectra shown in blue, eraser method spectra in green and destructive method spectra in purple.

Despite the promise of non-destructive ZooMS analyses, there are currently two significant obstacles reducing the efficacy of these approaches: (1) the lack of high molecular weight peptides (HMWP) and therefore limitations in providing highly resolved identifications; and (2) the current gaps in collagen reference data (applicable to all ZooMS analyses). While the observed peak intensity tended to be lower in the non-destructive methods, our analysis indicated that the non-destructive methods can produce peaks of similar quality to the traditional approach (Fig. 4), although the HMWP are typically more highly resolved in spectra derived from destructive analysis (see Fig. 4, SI Table 2, and SI Figs 4 and 5). The low signal to noise ratio encountered with HMWP can be problematic for more highly resolved identifications as it is precisely these peptides that are often used to discriminate between closely related species (e.g. distinguishing between various deer species which have distinct peptides at m/z 3017 + 3033 (red and fallow deer), 3043 + 3059 (roe and white-tailed deer) and 3093 (reindeer) (Table 2)). Further testing is still needed to confirm the average peptide mass range that can be obtained and whether the resolution of the higher mass peptides could be improved, however it is likely that the lack of HMWP is due to degradation resulting in breakage of the longer peptides. As the bag technique is essentially removing loose strands of collagen from the exterior surface of the object, it is reasonable to assume that the majority of these strands are already somewhat damaged. Thus, the greater the level of damage, the lower the likelihood of finding intact longer peptides. Additionally, the overall quality of the collagen preserved in a given sample will likely be key in determining whether or not the bag method can be successfully applied. In highly degraded samples it can be difficult to obtain identifications using destructive ZooMS. As such, it is unlikely that the bag method would produce reliable identifications for samples containing limited intact collagen, either as a result of age-related degradation or other taphonomic or storage conditions.

Although the destructive ZooMS method provides the greatest success rate and highest level of resolution, there are significant advantages to the non-destructive methods. Most obvious is the limited intervention, particularly when using the bag method as the artefacts themselves need not be removed from their storage location, and it requires no destructive sampling or even handling of the artefact (other than being transferred to a new storage bag). While the level of resolution currently achievable with the bag method may not provide species-specific identifications in all instances, it can provide an indication of whether or not further, potentially destructive analysis would be a worthwhile risk. For example, if the bag method indicates an unexpected genus, curators and collection managers who were previously (understandably) reluctant to allow physical sampling of the objects in their care may be more inclined to consider additional forms of analysis.

The results of the eraser method would appear to suggest that this method can work quite well on archaeological bone samples. However, in the first instance, the bag method may be advisable, as it has little to no effect on the integrity or appearance of the artefact. While the eraser method is non-invasive in that no visible amounts of bone are being removed, it can alter the appearance of an artefact by essentially cleaning the sampled area, potentially affecting the integrity of the artefact; the bag method eliminates this issue from the equation completely.

Some precautions do need to be considered when using the proposed bag method. The most significant issue is that any bags tested can only have been used once and for a single sample. If multiple samples are stored in the bag at the same time, or if the bag has been reused, unambiguous identification of the object(s) would not be possible. However, the forced bag method, using a clean new bag, can overcome this issue. Additionally, as with the eraser method, contaminant peptides, such as keratin, are much more prevalent than in the destructive method (SI Fig. 4). While precautions are always taken to limit contaminants (such as wearing gloves when handling artefacts and cleaning surfaces/tools between samples, etc.), keratin is a common lab contaminant as it is found in hair, nails and skin. This also means, however, that its peptide masses are well documented and can therefore be easily identified in a spectrum and excluded from further analysis. Similarly, there are also several unidentified peaks in a number of the bag spectra that likely result from plastic residues leaching out of the bag (SI Fig. 5). These however, tend to appear at the lower end of the m/z spectra where few peaks are regularly used for identification, and they can generally be recognized by the pattern of equally spaced repeating peaks. Thus, care needs to be taken when analyzing the spectra to ensure that the peaks applied for identification are in fact true collagen markers.

The desire to elucidate an artefact’s origins and significance, while simultaneously maintaining its integrity, is a dilemma with which archaeologists and collections curators are all too familiar. ZooMS is an ideal tool for identifying worked bone artefacts which have been stripped of all diagnostic anatomical features; however the need for (even minimally) destructive sampling is problematic for rare or culturally significant objects. The newly developed non-destructive approaches showcased in this study can provide greater insight into the symbolic nature of bone artefacts while preserving them for future study and exhibition. Furthermore, this study demonstrated how systematic analysis of worked bone artefacts has the potential to reveal quite unexpected links between the tangible material objects and their intangible cultural significance.

## Materials and Methods

### ZooMS analysis

#### Original bag method

The points were transferred to new zip-seal storage bags and 2 mL of 0.6 M HCl was added to each of the original storage bags, as well as to one new bag which served as a blank control. The bags were heated at 65 **°**C for 4 hours, and the acid was pipetted out of the bags and neutralized using 0.1 M NaOH. The neutralized solution was freeze-dried to reduce the volume, and then re-suspended in 50 μL of 50 mM ammonium bicarbonate buffer (NH_4_HCO_3_, pH 8.0, AmBic); 0.4 μg of trypsin was added and the samples were heated at 37 **°**C for approximately 18 hours. The samples were acidified to 0.1% trifluoroacetic acid (TFA) and the collagen peptides extracted using 100 μL C18 resin ZipTip® pipette tips (EMD Millipore). Samples were spotted in triplicate, along with calibration standards, onto a Bruker ground steel target plate using 1 μL each of extracted collagen and matrix solution (α-cyano-hydroxycinnamic acid). MALDI-ToF-MS was performed on the samples using a Bruker Ultraflex III mass spectrometer. Spectra were analyzed using mMass software^48^ and the resultant averaged spectrum for each sample identified by comparing them with published data^19,49,50^.

#### Forced bag method

A second ‘forced’ bag method was developed in an attempt to both improve the clarity of the spectra and to rule out potential contamination resulting from the re-use of storage bags. Following the transfer of the artefacts to previously unused zip-seal sample bags (Bryson Packaging Ltd., UK), they were gently rubbed within the bags for several minutes to replicate long term storage. Longer periods of rubbing (i.e., 5 minutes time, or several times over a day) were also tested, as well as leaving the samples in the new bags over several days prior to analysis; neither of these modifications, however, appeared to have a significant impact on the resulting spectra. The artefacts were transferred to new storage bags, and 1–2 mL of AmBic was added to the forced bags and to an additional empty bag as a control blank. The bags were heated at 65°C for 4 hours, then the AmBic was pipetted out of the bags and the volume reduced in a centrifugal evaporator. Samples were re-suspended in 50 μl of AmBic and the methods for enzyme cleavage, purification and MALDI-ToF-MS followed the same procedure as above.

#### Eraser method

Following the method described by Fiddyment *et al*.^14^, a small section of PVC eraser was cut for each artefact. The eraser was rubbed over an area of the sample several times and the eraser bits collected. 75 μL of AmBic was added to the eraser pieces along with 0.4 μg of trypsin, and then heated for 4 hours at 37°C. The samples were acidified, purified, and subjected to MALDI-ToF-MS in the same manner as described above

#### Destructive ZooMS

Destructive ZooMS followed a slightly modified procedure to that described in Buckley *et al*.^19^. Briefly, 10–30 mg of bone was subsampled and placed in 250 μL of 0.6 M HCl at 4 °C until demineralised. The acid was discarded and the sample rinsed with 200 μL of 0.1 M NaOH to remove humics and other chromophoric compounds. Samples were then rinsed three times in 200 µL of AmBic, and gelatinised at 65 °C for 1 hour in 100 μL of AmBic. 0.4 μg of trypsin was added to 50 μl of the supernatant and samples were heated at 37 °C for approximately 18 hours, then acidified, purified, and subjected to MALDI-ToF-MS in the same manner as described above.

#### Ancient genomic analysis

Ancient DNA analyses were performed in dedicated facilities at the University of York (detailed methods are found in Supplementary Information S1). Bone points were subsampled with a dremel, chemically decontaminated, and DNA extracted following a modified silica-spin protocol^51,52^. To resolve the bear species identification of DR-21 and DR-1662, we amplified a 217 bp fragment of the cytochrome b gene spanning positions 15311–15528 of the *Ursus arctos* mitochondrial genome (Genbank accession NC003427). Resultant sequences were edited, and species and haplotype identifications were confirmed through multiple alignments in BioEdit^53^ with 75 previously published bear sequences of extant and extinct American bear species^30,54–56^. The ancient mtDNA sequences were deposited in Genbank under Accessions MG696868-MG696869.

DNA extracts from five samples were converted into double-stranded Illumina sequencing libraries^57,58^ then pooled in equimolar concentrations and single-end sequenced (SE80; SE100) on a HiSeq2500 Illumina platform at the National High-throughput DNA Sequencing Centre, University of Copenhagen, Denmark. Sequencing results are presented in SI Table 1; sequencing datafiles for the bone points, extraction blanks and library controls are available through the European Nucleotide Archive under Accession PRJEB23998.

The raw reads were quality filtered and trimmed of adaptors using cutadapt v1.11^59^. FastQ Screen (http://www.bioinformatics.babraham.ac.uk/projects/fastq_screen) was used for initial species identification, aligning to the human (hd37d5), red deer/elk (*Cervus elaphus hippelaphus* GCA_002197005.1 Celaphus1.0) and polar bear (*Ursus maritimus* GCF_000687225.1) genomes. The individual reads from the human and bear bone points were mapped to the human (hg19) and polar bear genomes (GCF_000687225.1) Authentication of the ancient DNA sequences was undertaken through the assessment of post-mortem degradation^60^. Molecular sex identification was undertaken using the method proposed in Skoglund *et al*.^61^. HaploFind^62^ was used to identify defining mutations and assign mitochondrial haplogroups. Ancestry of the human bone points was conducted with LASER version 2.04^63^ through comparison with a reference panel of 650 K SNPS from 938 modern humans.

## Supplementary information

Supporting Information

Supplementary Datafiles

## Data Availability

The genetic sequences reported in this paper have been deposited in the GenBank database (accession nos MG696868-MG696869) and through the European Nucleotide Archive (Accession PRJEB23998). The MS datasets generated and analysed for this study are available as Supplementary Data Files.
