# Supplementary material for: Identifying Archaeological Bone via Non-Destructive ZooMS and the Materiality of Symbolic Expression: Examples from Iroquoian Bone Points
Source: Sci Rep. 2019 Jul 30;9:11027. doi: 10.1038/s41598-019-47299-x (PMC6667708; doi:10.1038/s41598-019-47299-x)
Supplement: Supplementary file 1 — Supporting Information [file 41598_2019_47299_MOESM1_ESM.pdf]

Supporting Information for manuscript:

Identifying Archaeological Bone via Non-Destructive ZooMS and the Materiality of Symbolic Expression: Examples of Iroquoian Bone Points

Krista McGrath*[1]; Keri Rowsell[1,2]; Christian Gates St-Pierre[3]; Andrew Tedder[4]; George Foody[5]; Carolynne Roberts[1]; Camilla Speller[1,6]; Matthew Collins[1,7]

[1] BioArCh, Department of Archaeology, University of York, York, UK
[2] London School of Economics and Political Science, London, UK
[3] Département d'anthropologie, Université de Montréal, Montréal, Québec, Canada
[4] School of Chemistry & Bioscience, University of Bradford, Bradford, UK
[5] School of Applied Sciences, University of Huddersfield, Huddersfield, UK
[6] Department of Anthropology, University of British Columbia, Vancouver, Canada
[7] EvoGenomics, Natural History Museum of Denmark, University of Copenhagen, Copenhagen, Denmark

* corresponding author: krista.mcgrath@york.ac.uk

Supporting Information

S1: DNA Sampling and Extraction

Ancient DNA was performed to further confirm the ZooMS results and resolve species identifications for five samples: two bear samples (DR-21 and DR-1662) and three human samples (DR-894s, DR-1797s and MC-398s). Ancient DNA extraction was performed in dedicated facilities at the University of York. In order to minimize destructive sampling, a dremel was used to cut a small portion of each point. The subsampled bone was decontaminated through submersion in 6% NaClO for 5 min, rinsed three times in ddH_2O, and exposed to UV irradiation for 10 min on two sides. The bone samples were then ground into powder and 100-150 mg was weighed out for DNA extraction. The samples first underwent a pre-digestion step to remove exogenous DNA[1]: samples were incubated in a rotating oven at 37°C for 18-24 hours in 1 mL of lysis buffer (EDTA, pH 8.0; 0.5 mg/mL Proteinase K). The samples were then removed from the oven, centrifuged for 3 min, and the supernatant was removed. A subsequent 1.5 mL of lysis was added to the remaining bone powder and returned to the rotator oven for 18-24 hours at 50°C. DNA was extracted following a silica-spin column protocol[2] modified as in Yang et al.[3] Briefly, 1.2 mL of lysis was concentrated down to approximately 150 µL in an Amicon Ultra-4 Centrifugal Filter (10 kDa, Millipore). 5X PB buffer (Qiagen) was added to the concentrated lysis in the Amicon, resuspended then transferred to a Qiagen MinElute PCR purification column. The samples were centrifuged at 7000 rpm for 1 min and the flow-through discarded. 500 µL of PE buffer (Qiagen) was added and the column centrifuged at 7000 rpm for 1 min and the flow-through discarded. A second 500 µL of PE was added then centrifuged at 13000 rpm for 1 min and the flow-through discarded. The columns were spun for 1 min at 13000 rpm to remove residual ethanol. DNA was eluted in two aliquots of 25 µL of EB. For sample DR-1797s, DNA was extracted from both pre-digestion and second digestion fractions.

S2: mtDNA PCR Amplifications and Sequencing

Mitochondrial DNA Analysis

Mitochondrial DNA (mtDNA) fragments were targeted via simplex PCR to further resolve the species identifications of the bear points. For DR-21 and DR-1662, we amplified a 217 bp fragment of the cytochrome *b* gene using forward primer UR-F6 (5' CAACATCCGAAAAACCCACCC 3') and UAM-R223 (5' AGTGAACATCTCGGCAAATATGGG 3') spanning positions 15311-15528 of the *Ursus arctos* mitochondrial genome (Genbank accession NC003427). Amplifications were performed in a 30 µL reaction containing nuclease free H2O, 1.5X of GeneAmp® 10X PCR Gold Buffer, 2.5 mM MgCl2 , 0.2 mM dNTP, 1 mg/ml BSA, 0.3 µM of each primer, 2.5 U of AmpliTaq® Gold LD DNA Polymerase (Applied Biosystems) and 3 µL of DNA extract. Amplification conditions involved an initial denaturation step of 95°C for 12 min, followed by 60 cycles of 52°C for 30 sec (annealing), 94°C for 30 sec (denaturing), and 72°C for 90 seconds (elongation), followed by a final annealing step of 72°C for 5 min. Amplified samples were visualized on a 2% agarose gel and sequenced at Eurofins UK. Resultant sequences were edited with ChromasPro to remove primer portions. Sequences were submitted to Genbank BLAST for initial identifications, and species and haplotype identifications were confirmed through multiple alignments in BioEdit[4]. The cytochrome *b* sequences from DR-21 and DR-1662 were aligned with 75 previously published bear sequences of extant and extinct American bear species[5–8]. The sequences from the two bone points both matched identically with each other and *U. americanus* A-East cytochrome b

haplotype (Accession KM257060) [8]. The ancient mtDNA sequences were deposited in Genbank under Accessions MG696868-MG696869. No amplifications were observed within any of the blank extractions or negative controls run alongside the ancient samples.

S3: Library Creation and Illumina Sequencing

DNA extracts from five samples (DR-21, DR-1662 [bears], DR-894s, DR-1797s and MC-398s [humans]) were converted into double-stranded Illumina sequencing libraries for shotgun sequencing following the protocol by Meyer and Kircher[9] and modified according to Fortes and Paijmans[10]. Libraries were prepared using 25 µL of DNA, and individual P5 and P7 barcodes were included for each sample. For sample DR-1797s, libraries were prepared from both the pre-digestion and second digestion DNA extracts. 3 µL of the resulting libraries were amplified and indexed in a 25 µL reaction containing 1X AmpliTaq Buffer, 2mM $MgCl_2$, 0.1 mg/ml BSA, 0.25mM dNTPs, 1.25U AmpliTaq Gold 360, 0.2µM IS4 Forward Primer and 0.2µM each of individually barcoded P7 Indexing Primer. Amplification thermal cycling conditions were as follows: 10 min at 94°C; between 15 and 19 cycles of 30 sec at 94°C, 45 sec at 60°C and 45 sec at 72°C, with a final extension step of 5 min at 72°C. Amplified libraries were purified using Qiagen MinElute PCR purification columns, quantified using a Qubit 2.0 Fluorometer, and quality assessed on an Agilent 2100 Bioanalyzer using a High Sensitivity Chip. Libraries were constructed from blank extracts, and negative controls were processed along with all samples to monitor for contamination. Indexed libraries were pooled in equimolar concentrations (along with blanks and negative controls) and single-end sequenced (with read length 80bp and/or 100bp) on a HiSeq2500 Illumina platform at the National High-throughput DNA Sequencing Centre, University of Copenhagen, Denmark. The library from DR-894s was sequenced twice (SE80 and SE100); data from both sequencing runs was combined before adaptor trimming and quality filtering. Sequencing results are presented in SI Table 1. Sequencing datafiles for the bone points, extraction blanks and library controls are available through the European Nucleotide Archive under Accession PRJEB23998.

S4: Data Processing and Read Mapping

The raw reads obtained from the sequenced libraries were trimmed for adapter and P5 index sequences using cutadapt v1.11. During P5 index trimming, one error in the index sequence was allowed (parameter −e 0.125). The reads were filtered to a minimum phred-scaled quality score of 20 (-q 20) and any sequence less than 30 bp in length (-m 30) or that did not match the correct P5 index was discarded from analysis. FastQ Screen (http://www.bioinformatics.babraham.ac.uk/projects/fastq_screen) was used for initial species identification, aligning the trimmed reads to three genomes: human (hd37d5), red deer/elk (*Cervus elaphus hippelaphus* GCA_002197005.1 Celaphus1.0) and polar bear (*Ursus maritimus* GCF_000687225.1). The FastQ Screen results confirmed the human genome as the most likely source of sequences for DR-894s and DR-1797s (Supplementary Figure 1). Likewise, the bear genome was confirmed as the most likely source of sequences for DR1662, and DR21, although the latter had <0.5% of the sequences mapping to any genome. No species identity, however, could be confirmed for MC-398s due to a lack of aligned reads.

The individual reads from the human bone points were then mapped to the human reference genome (hg19) using the Burrows-Wheeler Alignment 0.7.5a[12] with the following parameters (bwa aln -l 16500 - n 0.02 -o 2). Similarly, the individual reads from the bear bone points were

mapped to the polar bear genome (*Ursus maritimus* GCF_000687225.1) using the same parameters. Reads were sorted and PCR duplicates removed using Samtools v0.1.19[12]; mean fragment lengths were estimated using BAMStats (http://bamstats.sourceforge.net/). The percentage of endogenous DNA within all five bone points was extremely low. The human points DR-894s, DR-1797s and MC-398s displayed 2.94%, 2.19% and 0.01% of the sequences mapping to the human genome, respectively. As noted above, two separate DNA extracts were analysed from DR-1797s: the 'pre-digestion' fraction and the second digestion fraction. The library sequenced from the pre-digestion fraction yielded a greater quantity of endogenous DNA (2.19%) compared to the second digestion (0.27%) and thus, the data from former was used for subsequent genomic analysis. As noted from the FastQ Screen results, MC-398s had very few sequences mapping to the human genome (< 2000), and was excluded from further genomic analysis. The bear bone points displayed 0.06 % and 12.71% of sequences mapping to the polar bear genome for DR-21 and DR-1662, respectively (SI Table 1).

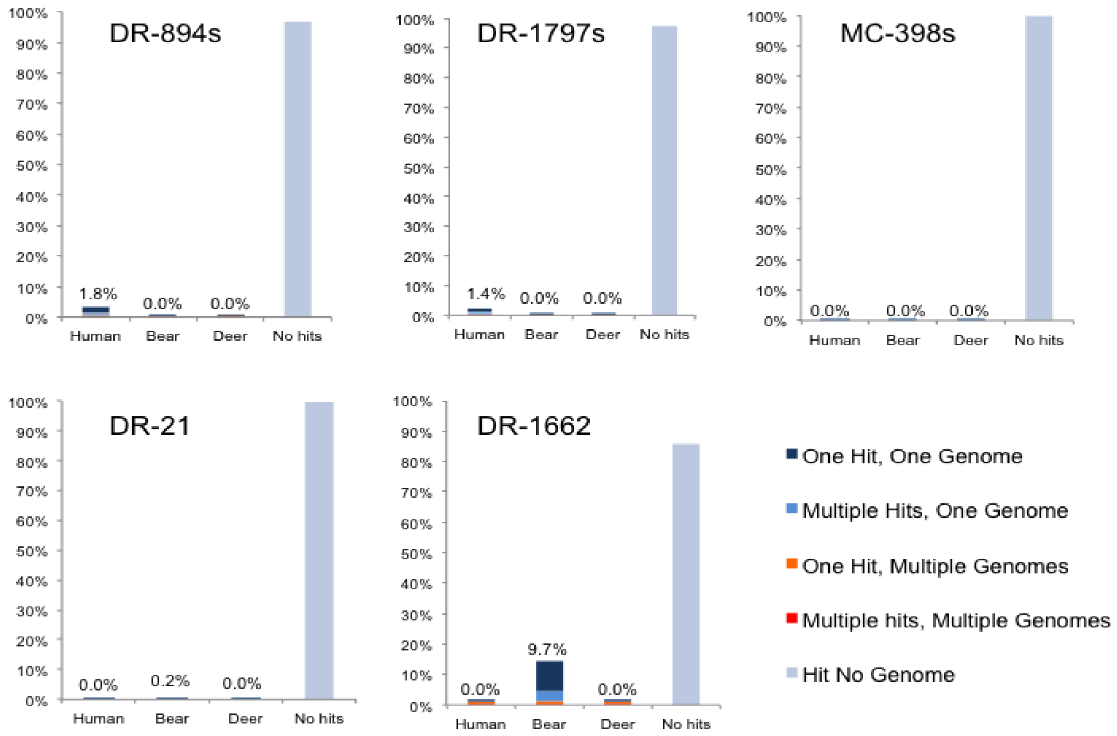

SI Figure 1: Histograms illustrating the relative frequency of raw sequence read alignments to the human, red deer and polar bear genomes for the five bone points. Data labels indicate the percentage of sequences mapping uniquely to a single genome.

S5: Genomic Analysis

Data Authenticity

Authentication of the ancient DNA sequences was undertaken through the assessment of post-mortem degradation including short sequence length and characteristic misincorporation patterns, particularly the deamidation of cytosine (C-T) at the 5' ends of molecules[13]. Sequence length distributions and damage patterns were assessed through the mapDamage2.0 package

(Jónsson et al., 2013) (SI Figure 2). The two bear samples (DR-21 and DR1662) and the two human bone point samples with over 1% endogenous DNA (DR-894s and DR-1797s) displayed average read lengths of 71 bp. An increase in C to T and G to A changes towards the 5' and 3' ends of reads was observed across all four samples, with the C to T misincorporation at the 5' read end ranging from 6.7-16.0% and complementary G to A frequencies ranging from 6.1-11.0% (SI Figure S2).

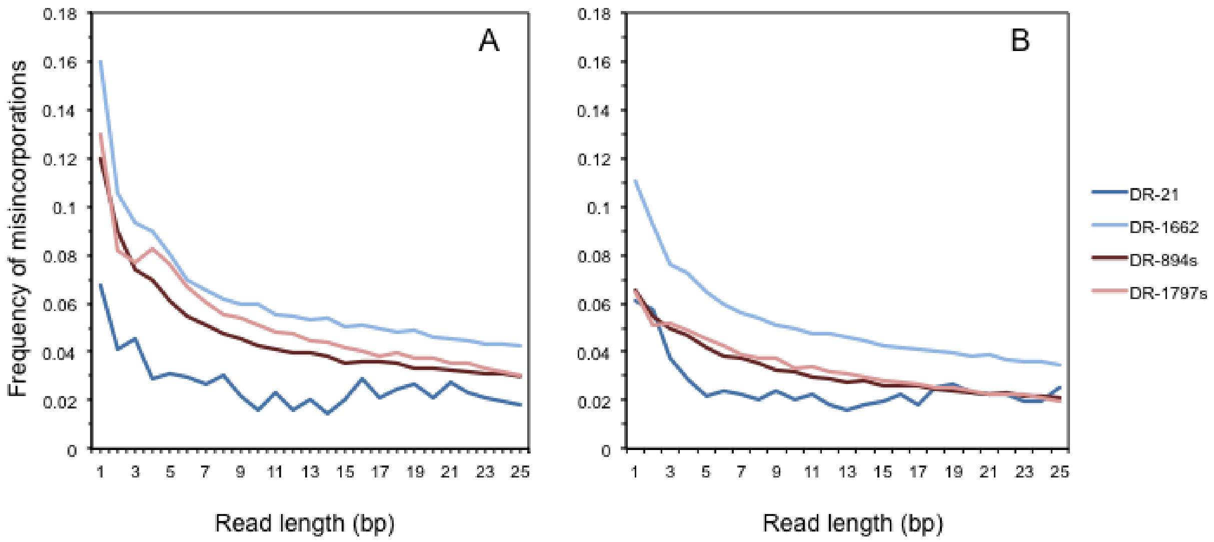

SI Figure 2. Misincorporation patterns for the bear (DR-21 and DR-1662) and human (DR-894s and DR-1797s) bone points' genomic sequences. (A) Frequencies of 5' C to T transitions; (B) frequencies of 3' G to A transitions.

Molecular Sex Determination

Sex identification was undertaken on two human bone point libraries (DR-894s and DR-1797s) by calculating the ratio of reads aligning to the Y chromosome to reads aligning to both sex chromosomes (Ry)[14]. The 95% confidence interval (CI) was computed as Ry ± 1.96 × Ry × (1 - Ry)/(Nx + Ny), where Nx and Ny are the total number of reads aligning to the X chromosome and Y chromosome respectively. Using this method, both bone points could be identified as deriving from male individuals (SI Table 1).

Mitochondrial Genome Analysis

To determine mitochondrial haplogroups of the human bone points, we aligned the trimmed reads to the revised Cambridge Reference Sequence (rCRS) using mapping parameters described in S4, and a minimum mapping quality of 30 and called consensus mitochondrial genome sequences using Samtools mpileup (with parameters -B and -Q 30) and vcfutils.pl (vcf2fq). HaploFind[15] was used to identify defining mutations and assign sample haplogroups. Only one of the bone points (DR-894s) produced sufficient sequences (>1,000 mtDNA sequences) to obtain a confident mtDNA haplotype, corresponding to haplogroup C1, one of the major founding haplogroups of the Americas[16,17].

Principal Components Analysis

PCA was performed using LASER version 2.04[18] on the two bone point samples identified as human via FastQ Screen. This comprises a two-step process in which modern reference samples and ancient samples are computed independently. In the first step, data comprising approximately 650K SNPS from 938 modern humans (HDGP_938) [19] is used to create a reference space by performing a standard PCA. In the second step, the ancient samples are mapped into the reference space by simulating sequencing data for each reference individual that matches the coverage pattern of the ancient sample. A second PCA is then performed, which includes both simulated reference data and the ancient samples. Finally, Procrustes analysis was used to project the results of the ancient sample PCA into the modern sample reference space. Due to the low endogenous DNA content of the bone samples, relatively few SNPs could be called for the two bone points (DR-894s 7585 SNPs; DR1797s 3094 SNPs, Figure 4 main text).

SI Table 1. Single-end Illumina sequencing results for samples DR-894s, DR-1797s, MC-398s, DR-21s and DR-16

Bone Point Name	Sample Name	Library ID	P5 Barcode	ENA Accession	Total Sequenced Reads	Reads After Adaptor Trimming and Quality Filtering	Unique Mapped Reads	% Endogenous DNA	Mean Fragment Length (bp)	R_y	95% CI	Genetic Sex Assignment	Total Mapped mtDNA Sequences	Haplogroup Assignment
DR-894s	13054	13054 (3QLY)	ACGAACTT	ERS2059530	18,997,251	18,839,315	553,894	2.94	72	0.2272	0.2211-0.2333	XY	1174	C1c
DR-894s	13054	13054 (JPEE)	ACGAACTT	ERS2059531										
DR-1797s	13061	13061	CCATCTTA	ERS2059532	10,630,048	10,175,368	27,451	0.27	71	-	-	-	-	-
DR-1797s	13061	13061L	CGATCGGA	ERS2059533	10,709,556	10,463,067	228,847	2.19	68	0.1446	0.1367-0.1525	XY	190	N/A
MC-398s	13065	13065	TGGCGTTA	ERS2059534	13,000,031	12,846,159	1,610	0.01	63	-	-	-	-	-
DR-21s	13052	13052	AACCAGAA	ERS2059535	6,450,663	6,356,076	3,784	0.06	61	-	-	-	-	-
DR-1662s	13060	13060	AGTTGAAC	ERS2059536	4,846,202	4,786,647	577,694	12.07	81	-	-	-	-	-
Extraction blank	eBKH_010	eBKH_010	AACGAAGT	ERS2059537	1,285,247	326,283	323	0.10	63	-	-	-	-	-
Extraction blank	eBK1_004	eBK1_004	AACCGAAC	ERS2059538	1,900,167	76,044	182	0.24	64	-	-	-	-	-
Library blanks	LBL_710	LBL_710	TTGGCAGA	ERS2059539	2,249,246	775,857	175	0.02	62	-	-	-	-	-

S6: ZooMS Bag Method Contamination Control

Two contamination issues commonly arose with the bag extraction method, those being co-extraction of non-endogenous peptides (often keratin, see SI Figure 4) and plastic residues from the storage bags (see SI Figure 5). However, contaminants can be easily recognized and removed from downstream analysis. All bag spectra were checked against a list of known contaminant peptide masses (such as keratin and porcine trypsin), and those peaks were then excluded from further analysis.

Keratin, the main structural component of hair, nails and the outer layer of skin, is a common laboratory contaminant and was the main source of contamination in the bag spectra. Being a common contaminant, the masses for human keratin peptides are widely available and any peaks matching with known keratin masses were excluded from further analysis. The keratin peaks also tend to be easily identifiable in the spectra as their intensity is generally greater than the actual sample collagen (SI Figure 4). The plastic residues pose less of a risk in terms of identification error as they generally form a distinct wave pattern of repeating peaks, usually 14 m/z apart and appearing at the lower molecular weight end of the spectra (SI Figure 5).

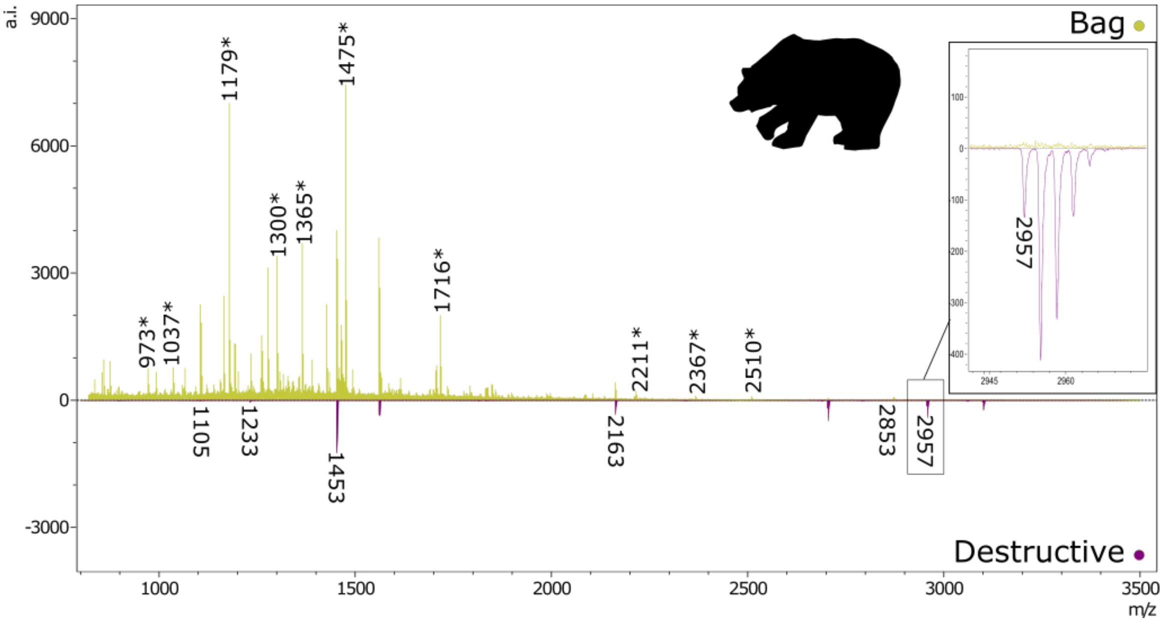

SI Figure 4 Comparison of MALDI-TOF-MS spectra from the bag and destructive ZooMS methods for sample DR-491s depicting keratin contamination (all masses with *) and the lack of high molecular weight peaks in the bag extraction method. Peaks labelled in the destructive spectra are those used to identify the sample as bear; these peaks also appear in the bag spectra, however only keratin peaks have been labelled.

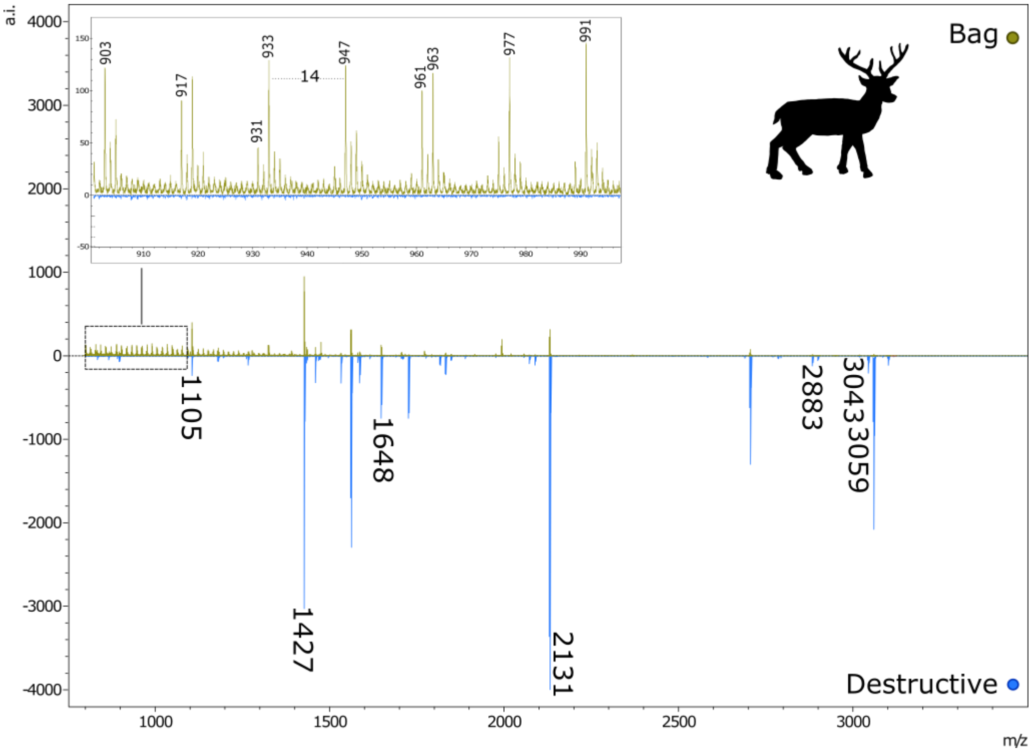

SI Figure 5 Comparison of MALDI-TOF-MS spectra from the bag and destructive ZooMS methods for sample DR-1588s depicting likely plastic residue contamination in the bag extraction method. The lack of high molecular weight peaks in the bag extraction can also be seen. Peaks labelled in the destructive spectra are those used to identify the sample as white tailed deer.

SI Table 2. *m/z* markers used to make ZooMS identifications for each ZooMS method tested.

Sample	Method	ZooMS ID	P1	A1	A2	B	C	P2	D	F1	F2	G1	G2
DR-21s	Original bag	Bear	1105	x	1233	1453	1566	x	2163	2853	x	X	x
	Forced bag	N/T	-	-	-	-	-	-	-	-	-	-	-
	Eraser	Probable bear	1105	x	x	1453	1566	x	2163	2853	x	X	x
	Destructive	Bear	1105	x	1233	1453	1566	x	2163	2853	x	2957	x
DR-491s	Original bag	Carnivore - probable bear/cat	1105	x	x	? 1453	? 1566	x	? 2163	x	x	X	x
	Forced bag	Probable bear	1105	x	x	1453	1566	x	2163	? 2853	x	X	x
	Eraser	Probable bear	1105	x	x	1453	1566	x	2163	x	x	X	x
	Destructive	Bear	1105	x	1233	1453	X	x	2163	2853	x	2957	x
DR-894s	Original bag	No ID	-	-	-	-	-	-	-	-	-	-	-
	Forced bag	Human	1105	x	1235	1477	1580	x	2115	2869	x	? 2957	x
	Eraser	Human	1105	x	x	x	1580	x	2115	2869	x	x	x
	Destructive	Human	1105	x	1235	1477	1580	x	2115	2869	? 2885	2957	x
DR-1044s*	Eraser	No ID	-	-	-	-	-	-	-	-	-	-	-
DR-1130s	Original bag	No ID	-	-	-	-	-	-	-	-	-	-	-
	Forced bag	No ID	-	-	-	-	-	-	-	-	-	-	-
	Eraser	Carnivore - probable bear/cat	1105	x	x	1453	1566	x	? 2163	x	x	x	x
	Destructive	Bear	1105	x	1233	1453	? 1566	x	2163	2853	? 2869	2957	? 2973
DR-1454s	Original bag	Bovid/Cervid	1105	x	x	1427	1580	1648	x	2883	x	x	x
	Forced bag	Bovid/Cervid	1105	x	x	1427	1580	1648	x	2883	x	x	x
	Eraser	Bovid/Cervid	1105	x	x	1427	1580	1648	2131	2883	x	x	x
	Destructive	White-tailed deer	1105	x	x	1427	x	1648	2131	2883	x	3043	3059
DR-1466s	Original bag	No ID	1105	x	x	? 1427	x	x	x	x	x	x	x
	Forced bag	Bovid/Cervid	1105	x	x	1427	1580	1648	2131	x	x	x	x
	Eraser	Bovid/Cervid	1105	x	x	1427	1580	1648	2131	x	x	x	x
	Destructive	White-tailed deer	1105	1180	1196	1427	1580	1648	2131	2883	2899	3043	3059

DR-1588s	Original bag	No ID	-	-	-	-	-	-	-	-	-	-	-
	Forced bag	Bovid/Cervid	1105	x	x	1427	1580	1648	2131	2883	? 2899	x	? 3059
	Eraser	Bovid/Cervid	1105	x	x	1427	1580	1648	2131	x	x	x	x
	Destructive	White-tailed deer	1105	1180	1196	1427	1580	1648	2131	2883	2899	3043	3059
DR-1662s	Original bag	Probable bear	1105	x	1233	1453	1566	x	2163	2853	x	x	x
	Forced bag	Bear	1105	x	x	1453	1566	x	2163	2853	x	? 2957	x
	Eraser	Bear	1105	x	x	1453	1566	x	2163	2853	x	x	x
	Destructive	Bear	1105	x	1233	1453	X	x	2163	2853	x	2957	x
DR-1797s	Original bag	No ID	-	-	-	-	-	-	-	-	-	-	-
	Forced bag	Human	1105	x	1235	1477	1580	x	2115	x	x	x	x
	Eraser	Human	1105	x	x	x	1580	x	2115	x	x	x	x
	Destructive	N/T	-	-	-	-	-	-	-	-	-	-	-
DR-1926s	Original bag	No ID	-	-	-	-	-	-	-	-	-	-	-
	Forced bag	Possible carnivore	1105	-	-	1453	-	-	-	-	-	-	-
	Eraser	Possible carnivore	1105	-	-	1453	-	-	2105	-	-	-	-
	Destructive	Probable unknown carnivore	? 1105	-	-	1453	-	-	2105	2853	-	-	-
DR-2271s*	Original bag	No ID	-	-	-	-	-	-	-	-	-	-	-
	Eraser	No ID	-	-	-	-	-	-	-	-	-	-	-
DR-5448s*	Original bag	No ID	-	-	-	-	-	-	-	-	-	-	-
	Eraser	No ID	-	-	-	-	-	-	-	-	-	-	-
MC-398s	Original bag	No ID	-	-	-	-	-	-	-	-	-	-	-
	Forced bag	Human	1105	x	1235	1477	1580	1619	2115	2869	x	2957	x
	Eraser	No ID	1105	x	x	x	X	x	? 2115	x	x	x	x
	Destructive	Human	1105	x	1235	1477	1580	1619	2115	2869	x	2957	x
BgFo-18	Original bag	No ID	-	-	-	-	-	-	-	-	-	-	-
	Forced bag	No ID	1105	-	-	-	-	-	-	-	-	-	-
	Eraser	Possible carnivore	1105	x	x	? 1453	X	x	x	x	x	x	x
	Destructive	Bear	1105	x	1233	1453	X	x	2163	2853	? 2869	2957	x

? Indicates peak is present but at low intensity, or below signal to noise threshold; “x” indicates no peak was present; “-“ indicates no useable peaks were identified in the spectra; “*” indicates the pendant; “**” indicates burnt samples. With the exception of the white-tailed deer, all *m/z* markers are from published sources[20–22]. The white-tailed deer *m/z* markers were determined from spectra obtained from a known reference specimen.

References:

1. Boessenkool, S. *et al.* Combining bleach and mild pre-digestion improves ancient DNA recovery from bones. *Mol. Ecol. Resour.* (2016). doi:10.1111/1755-0998.12623
2. Yang, D. Y., Eng, B., Waye, J. S., Dudar, J. C. & Saunders, S. R. Technical note: improved DNA extraction from ancient bones using silica-based spin columns. *Am. J. Phys. Anthropol.* **105,** 539–543 (1998).
3. Yang, D. Y., Liu, L., Chen, X. & Speller, C. F. Wild or domesticated: DNA analysis of ancient water buffalo remains from north China. *J. Archaeol. Sci.* **35,** 2778–2785 (2008).
4. Hall, T. A. BioEdit: a user-friendly biological sequence alignment editor and analysis program for Windows 95/98/NT. *Nucleic Acids Symp. Ser.* **41,** 95–98 (1999).
5. Krause, J. *et al.* Mitochondrial genomes reveal an explosive radiation of extinct and extant bears near the Miocene-Pliocene boundary. *BMC Evol. Biol.* **8,** 220 (2008).
6. Stone, K. D. & Cook, J. A. Phylogeography of black bears (Ursus americanus) of the Pacific Northwest. *Can. J. Zool.* **78,** 1218–1223 (2000).
7. Cronin, M. A., McDonough, M. M., Huynh, H. M. & Baker, R. J. Genetic relationships of North American bears (Ursus) inferred from amplified fragment length polymorphisms and mitochondrial DNA sequences. *Can. J. Zool.* **91,** 626–634 (2013).
8. Puckett, E. E., Etter, P. D., Johnson, E. A. & Eggert, L. S. Phylogeographic Analyses of American Black Bears (Ursus americanus) Suggest Four Glacial Refugia and Complex Patterns of Postglacial Admixture. *Mol. Biol. Evol.* **32,** 2338–2350 (2015).
9. Meyer, M. & Kircher, M. Illumina sequencing library preparation for highly multiplexed target capture and sequencing. *Cold Spring Harb. Protoc.* **2010,** db.prot5448 (2010).
10. Fortes, G. G. & Paijmans, J. L. A. Analysis of Whole Mitogenomes from Ancient Samples. *Methods Mol. Biol.* **1347,** 179–195 (2015).

11. Martin, M. Cutadapt removes adapter sequences from high-throughput sequencing reads. *EMBnet.journal* **17,** 10–12 (2011).
12. Li, H. & Durbin, R. Fast and accurate short read alignment with Burrows-Wheeler transform. *Bioinformatics* **25,** 1754–1760 (2009).
13. Jónsson, H., Ginolhac, A., Schubert, M., Johnson, P. L. F. & Orlando, L. mapDamage2.0: fast approximate Bayesian estimates of ancient DNA damage parameters. *Bioinformatics* **29,** 1682–1684 (2013).
14. Skoglund, P., Storå, J., Götherström, A. & Jakobsson, M. Accurate sex identification of ancient human remains using DNA shotgun sequencing. *J. Archaeol. Sci.* **40,** 4477–4482 (2013).
15. Vianello, D. *et al.* HAPLOFIND: a New method for high-throughput mtDNA Haplogroup assignment. *Hum. Mutat.* **34,** 1189–1194 (2013).
16. Schurr, T. G. *et al.* Amerindian mitochondrial DNAs have rare Asian mutations at high frequencies, suggesting they derived from four primary maternal lineages. *Am. J. Hum. Genet.* **46,** 613 (1990).
17. Tamm, E. *et al.* Beringian standstill and spread of Native American founders. *PLoS One* **2,** e829 (2007).
18. Wang, C., Zhan, X., Liang, L., Abecasis, G. R. & Lin, X. Improved ancestry estimation for both genotyping and sequencing data using projection procrustes analysis and genotype imputation. *Am. J. Hum. Genet.* **96,** 926–937 (2015).
19. Li, J. Z. *et al.* Worldwide human relationships inferred from genome-wide patterns of variation. *Science* **319,** 1100–1104 (2008).
20. Buckley, M. & Kansa, S. W. Collagen fingerprinting of archaeological bone and teeth remains from Domuztepe, South Eastern Turkey. *Archaeol. Anthropol. Sci.* **3,** 271–280 (2011).

21. Kirby, D. P., Buckley, M., Promise, E., Trauger, S. A. & Holdcraft, T. R. Identification of collagen-based materials in cultural heritage. *Analyst* **138,** 4849–4858 (2013).

22. Buckley, M., Collins, M., Thomas-Oates, J. & Wilson, J. C. Species identification by analysis of bone collagen using matrix-assisted laser desorption/ionisation time-of-flight mass spectrometry. *Rapid Commun. Mass Spectrom.* **23,** 3843–3854 (2009).
